# Epidemiologic profile of community-acquired *Clostridioides difficile* infections: a systematic review and meta-analysis

**DOI:** 10.1017/S0950268825000202

**Published:** 2025-03-04

**Authors:** Neri Alejandro Álvarez-Villalobos, Fernando Gerardo Ruiz-Hernandez, Ana Camila Méndez-Arellano, Jhoan Manuel Azamar-Márquez, Adrián Camacho-Ortiz

**Affiliations:** 1 Facultad de Medicina, Universidad Autónoma de Nuevo León, Av. Dr. José Eleuterio González 235, Mitras Centro, 64460 Monterrey, Nuevo León, México; 2 Centro de Análisis Avanzado de Información 360 (KER Unit México), Universidad Autónoma de Nuevo León, Av. Dr. José Eleuterio González 235, Mitras Centro, 64460 Monterrey, Nuevo León, México; 3 Knowledge and Evaluation Research Unit, Mayo Clinic, 210 2nd St SW, Rochester, MN 55905, USA; 4 Centro de Desarrollo en Investigación 360, Universidad Autónoma de Nuevo León, Av. Dr. José Eleuterio González 235, Mitras Centro, 64460 Monterrey, Nuevo León, México; 5 Servicio de Infectología, Facultad de Medicina y Hospital Universitario “Dr. José Eleuterio González,” Universidad Autónoma de Nuevo León, Monterrey, Nuevo León, México

**Keywords:** community-acquired infections, Clostridioides difficile infection, incidence, meta-analysis, prevalence

## Abstract

*Clostridiodes difficile*’s epidemiology has evolved over the past decades, being recognized as an important cause of disease in the community setting. Even so, there has been heterogeneity in the reports of CA-CDI. Therefore, the aim of this study was to assess the epidemiologic profile of CA-CDI.

This systematic review and meta-analysis were conducted according to PRISMA checklist and Cochrane guidelines (CRD42023451134). Literature search was performed by an experienced librarian from inception to April 2023, searching in databases like MEDLINE, Scopus, Web of Science, EMBASE, CCRCC, CDSR, and ClinicalTrials. Observational studies that reported prevalence, incidence of CA-CDI, or indicators to calculate them were included. Pool analysis was performed using a binomial-normal model via the generalized linear mixed model. Subgroup analysis and publication bias were also explored. A total of 49 articles were included, obtaining a prevalence of 5% (95% CI 3–8) and an incidence of 7.53 patients (95% CI 4.45–12.74) per 100,000 person-years.

In conclusion, this meta-analysis underscores that among the included studies, the prevalence of CA-CDI stands at 5%, with an incidence rate of 7.3 cases per 100,000 person-years. Noteworthy risk factors identified include prior antibiotic exposure and age.

## Introduction

Community-acquired *Clostridioides difficile* infection (CA-CDI) was first described in 1980. [1] In the past, it was thought that *C. difficile* was an exclusively hospital-acquired pathogen, but it is now recognized as an important cause of diarrhea in the community setting. CA-CDI can be defined, as per the Center for Disease Control and Prevention (CDC), as an onset of symptoms in the community or ≤ 48 h after admission to a healthcare institution, provided that the time of symptom onset was greater than 12 weeks after the last discharge from a healthcare institution. [2]

The epidemiology of *C. difficile* has evolved in the past decades, highlighting an increased transmission of CDI in community settings. [3, 4] The infection’s severity ranges from an asymptomatic colonization, mild to severe diarrhea, to life-threatening inflammation to the colon like a fulminant colitis that can lead to death. [5, 6] Approximately around 40% of patients with CA-CDI require hospitalization, 20% experience treatment failure, and about 28% have recurrent episodes. [7] Furthermore, CDI has a case-fatality rate of up to 14% within 30 days after diagnosis, with recurrences that can increase illness rates and decrease quality of life; still, morbidity and mortality could be determined by the changing virulence of the pathogen. [8–10]

CDI not only burdens patients and healthcare workers, but its impact is also noticeable in healthcare costs. CDI may have resulted in as much as $4.8 billion in excess healthcare costs in acute-care facilities during 2008. [11] Even so, CDI in the community might be underdiagnosed so the true burden of the disease might be greater than the ones reported by studies. [12] Still, a full appreciation of the burden that CDI has on the healthcare system is necessary for adequate resource allocation.

Even with the burden CA-CDI represents to the healthcare system, there has been considerable heterogeneity in the incidence and prevalence reports. Some studies state that there is a decline in cases of CA-CDI [13], while others point towards an increase of cases. [14, 15] Regardless, some studies that report either outcome compare the prevalence of CA-CDI to hospital acquired CDI (HA-CDI), yielding an inaccurate estimate of CA-CDI in the general population. Due to the heterogeneity of the reports and due to the increasing burden that CA-CDI cases are contributing to healthcare, the following systematic review and meta-analysis were developed, with the objective of assessing the epidemiologic profile of community-acquired *C. difficile.*

## Methods

This systematic review and meta-analysis were conducted according to the Preferred Reporting Items for Systematic Reviews and Meta-analyses (PRISMA) checklist and the guidelines from the Cochrane Handbook for Systematic Reviews of Interventions (Supplementary material I). This study was registered in the International Prospective Register of Systematic Reviews (PROSPERO) with the registration number CRD42023451134.

### Databases and search strategy

A comprehensive literature search was performed by an experienced librarian with the collaboration of the research team from inception up to April 2023. The search was conducted in multiple electronic databases including MEDLINE, Scopus, Web of Science, EMBASE, the Cochrane Central Register of Controlled Trials, Cochrane Database of Systematic Reviews, and Clinical trials.gov. The search included Medical Subject Headings (MeSH) terms as well as specific keywords related to the research question. A tailored search strategy was done in each database, with a combination of terms such as *C. difficile*, community acquired, prevalence, incidence, epidemiology. The following is the search strategy used for Web of Science: TS = (((("Clostridioides difficile" OR “*Clostridium difficile*” OR “Clostridium difficilis” OR “Pepto*clostridium difficile*” OR “Bacillus difficilis” OR “CA-CDI”) AND ("Community acquired" OR “community acquired infection” OR “community acquired disease” OR “community associated disease” OR “community associated infection” OR “Community-associated” OR “Community-acquired” OR community)) AND (prevalence OR “prevalence study” OR “incidence” OR “incidence rate” OR “rate, incidence” OR “epidemiology”))). The full tailored search for each database can be found in Supplementary material II.

### Searching and eligibility of studies

Retrieved articles were exported to EndNote reference software version 9 citation manager where they were deduplicated using the native deduplication function within the software, followed by manual review.

The studies that remained were imported into a systematic review software (Distiller SR), where the studies were screened in two phases: the title and abstract phase and the full-text phase. Articles included in both phases were evaluated independently by two reviewers. Studies included by at least one reviewer in the abstract screening phase were considered for full-text screening; this was done to increase sensibility in the included records.

During the full-text screening, agreement of inclusion between both reviewers was required for the study to be selected. Disagreements at any phase were resolved by consensus. Furthermore, before each phase, a pilot study was conducted to ensure inter-rater agreement by Kappa statistic. A Kappa statistic of >0.70 was set as an appropriate inter-rater agreement. The data extracted included the year of publication, country where the study was conducted, CA-CDI definition used by the authors, the number of samples processed, the diagnostic tool used, age groups included, population used to estimate incidence, CA-CDI cases reported, time frame, and risk factors reported by the authors.

### Eligibility criteria

Due to the nature of the outcome (prevalence and incidence), only published and unpublished cross-sectional or observational studies were considered for inclusion. The study population will be any primary study that reports the epidemiologic profile of *C. difficile*, specifically prevalence or incidence rates. If the studies do not report these indicators explicitly, they can be included if they provide other indicators that can be used to calculate prevalence or incidence rates.

### Outcome measurement of the study

The two main quantitative outcomes were the prevalence and incidence of CA-CDI, along with assessing qualitatively the factors associated with CA-CDI. The prevalence of CA-CDI was defined as the percentage of CA-CDI cases from a population of patients presenting diarrhea. Prevalence was extracted either as reported by the authors or the required information was calculated by the research team in the extraction sheet. Incidence was defined as the rate of new cases of CA-CDI over a specified time for the population at risk. Incidence was extracted as reported or calculated by dividing the new cases of CA-CDI reported by the result of multiplying the population at risk and the timeframe of the study in years. [16] Factors associated with CA-CDI were extracted as reported.

### Quality assessment

Two authors independently assessed the quality of the studies included. Depending on the study design, AXIS or New-Castle Ottawa (NOS) tools were used for cross-sectional and cohort or case-control studies respectively. [17, 18] For studies evaluated with AXIS, a predefined score of 17 of 20 for high-quality studies was set by the research team. On the other hand, for studies evaluated with New-Castle Ottawa, a predefined rating between 0–2, 3–5, and 6–9 was established as poor, fair, and good/high quality, respectively.

### Data processing and analysis

All the extracted data were recorded in a Microsoft Excel spreadsheet and cleaned for analysis. Heterogeneity in the data was expected; therefore, a random effects model was established as the primary model for the analysis a priori. We estimated the prevalence of CA-CDI in using a binomial – normal model for meta-analysis of prevalence via the generalized linear mixed model. [19] CA-CDI prevalence was reported as binomial proportion with 95% confidence intervals (CIs). CA-CDI incidence was also estimated with a generalized linear mixed model with summary findings being reported as CA-CDI cases per 100,000 person-years with 95% CIs. Statistical heterogeneity was tested using Cochran’s Q test and I^2^ index with its corresponding p-value. A statistical heterogeneity test with a p-value of less than 0.10 was considered significant for heterogeneity. [20] The values of I^2^ defined a priori as low, moderate, and high heterogeneity were 25%, 50%, and 75% respectively. [21] Pooled data are presented with forest plots.

Subgroup analysis, established a priori, by age groups was performed, and furthermore, sensitivity analysis was performed using influential analysis. Publication bias was explored by Egger’s test and visually with funnel plots. All statistical analyses were performed in R 4.3.0 with the meta and dmetar libraries. Factors associated with CA-CDI were qualitatively synthesized.

## Results

### Characteristics of the studies

**Figure 1.**
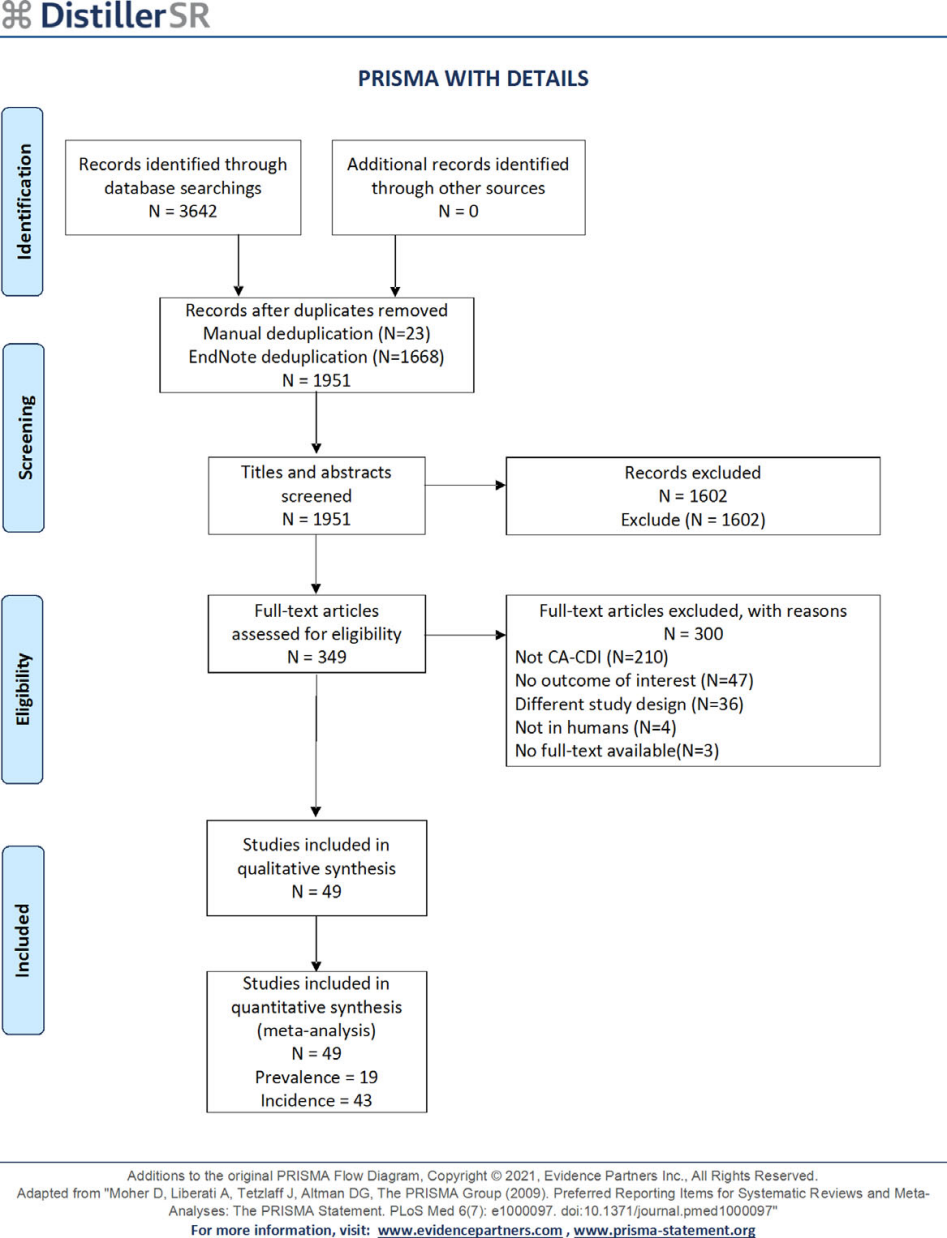
PRISMA flow diagram.

A total of 3,642 articles were retrieved on the initial search, from which 1,691 were excluded due to duplication. After title and abstract screening, 349 were included in full-text screening. After screening 49 articles in total, 19 articles were included for the prevalence outcome and 43 for the incidence outcome. A visual representation of the literature screening process can be seen in Figure 1.

#### Characteristics of the studies and study participants

Approximately 83,105 processed samples (not reported by all studies) for CDI were included in this study. Of the included articles, fifteen were from the USA [4, 22–35]; five from Spain [36–40]; four from Australia [71] [41–43]; three from Canada [3, 44, 45], Scotland [46–48], Sweden [49–51]; two from France [52, 53]; and one from Bailiwick of Jersey [54], China [55], Finland [8], Germany [56], Iceland [57], India [58], Iran [59], Ireland [60], Israel [61], Japan [62], Kuwait [63], Netherlands [64], New Zealand [65], and Slovakia [66]. The rest of the extracted characteristics can be seen in Table 1.

**Table 1.** Baseline characteristics of the included studies

Author (year)	Country	CA-CDI definition	Samples processed	Diagnostic tool	Age group	Population	CA-CDI cases	Timeframe in years
Kotila, et al. (2016)	Finland	Infection obtained outside a hospital, >4 weeks after hospital discharge, or < 2 days after admission	NR	TcdA, TcdB, culture of *C. difficile*, and NAATs	Mixed	5,500,000	10,643	6
Penit, et al. (2016)	France	Symptoms starting 8 weeks after hospital discharge or in the first 48 h of hospital stay	NR	ELISA, C-Dff Quik Chek Complete, *C. difficile* glutamate dehydrogenase, and toxin A/B antigens	Mixed	NR	27	0.58
Banks, et al. (2015)	Scotland	CDI case with onset of symptoms while outside a healthcare facility, and without discharge from a healthcare facility within the previous 12 weeks or with onset of symptoms within 48 h following admission to a healthcare facility without residence in a healthcare facility within the previous 12 weeks	NR	NR	>15 years old	1,601,849	158	1
Khanna, et al. (2016)	USA	Symptom onset occurred in the community or within 48 h of admission to a hospital, provided symptom onset was more than 12 weeks after the last discharge from a hospital.	NR	NR	Adults	308,745,538	263	8
Mori, et al. (2015)	Japan	Patient presenting in the outpatients setting with a diagnosis of CDI with no history of hospital discharge in the 12 weeks prior to the visit	208	*C. difficile* toxin assay or C Diff Quick Chek complete, isolates of *C. difficile* on stool culture and Immunocard CD toxin A & B	Adults and elderly	1,914,011	26	5
Alcalá, et al. (2015)	Spain	Patient with onset of symptoms while outside a healthcare facility, and without discharge from a healthcare facility within the previous 12 weeks or with onset of symptoms within 48 h following admission to a healthcare facility without residence in a healthcare facility within the previous 12 weeks	1800	CLO agar culture	Mixed	31,140,726	40	0.58
Jamal, et al. (2015)	Kuwait	Onset of symptoms occurring while the patient was outside a healthcare facility and the patient had not been discharged from a healthcare facility within 12 weeks before symptom onset, or the onset of symptoms occurring within 48 h upon admission to a healthcare facility and the patient had no prior stay in a healthcare facility within the 12 weeks prior to symptom onset	2,584	Presence of typical fluorescence color under UV light and API 20AN	Mixed	3,268,431	16	2
Lessa, et al. (2014)	USA	Positive *C difficile* specimen was collected as an outpatient or within 3 days after hospital admission and the patient had no documented overnight stays in a healthcare facility in the prior 12 weeks	NR	NAAT	Mixed	308,745,538	3,207	1
Taori, et al. (2014)	Scotland	CDI, which developed in a patient with no history of healthcare contact in the 12 weeks prior to diagnosis	NR	Toxin A and/or B by EIA and glutamate dehydrogenase, toxin A, and toxin B PCR17	Mixed	664,760	42	1
Slimings, et al. (2014)	Australia	Symptom onset in the community or < 48 h after admission to a hospital if symptom onset occurred >12 weeks after last discharge from a hospital	NR	Laboratory assay for *C. difficile* toxin A and/or B, or stool sample by culture or PCR.	Mixed	NR	1,320	2
Ingle, et al. (2013)	India	No history of past or present hospitalization in the past 12 weeks	150	*C. difficile* toxin (CDT) assay microscopy and culture, CDT A and B with ELISA	Mixed	NR	2	1.75
Gutiérrez, et al. (2013)	USA	Community acquired CDAD cases were individuals without in patient medical encounters in the twelve-week period prior to CDAD diagnosis	NR	NR	Adults	NR	1,018	13
Khanna, et al. (2012)	USA	Onset of symptoms occurred in the community or within 48 h of admission to a hospital, provided symptom onset was >12 weeks after the last discharge from a hospital	NR	NR	Mixed	123,310	157	15
Mitchell, et al. (2012)	Australia	Symptom onset was in the community or within 48 h of admission to a public hospital provided that symptom onset was more than 12 weeks after the last discharge from a healthcare facility in which skilled nursing care is provided, excluding residential aged care	NR	enzyme immunoassay or polymerase chain reaction detecting toxin A and/or toxin B, or culture	Mixed	NR	17 (in 2010) 54 (in 2011)	1
Vesteinsdottir, et al. (2012)	Iceland	If patients had not been hospitalized for the past 6 weeks, did not live in a nursing facility such as a nursing home or retirement home, and, if hospitalized, were diagnosed with CDI within 72 h from admission	1,693	CD toxin A and B by ELISA	Adults and elderly	318,452	30	1
Kuntz, et al. (2011)	USA	Diagnosis of CDI in the outpatient setting with no history of hospital discharge in the 12 weeks before diagnosis, or (2) a primary diagnosis upon hospital admission and no history of hospital discharge in the 12 weeks before diagnosis	NR	Primary or secondary diagnosis of ICD–9 code 008.45 for ‘Infection due to *Clostridium difficile*’ listed on an inpatient or outpatient insurance claim	Mixed	680,783	304	4
Allard, et al. (2011)	Canada	NR	NR		Mixed	1,850,000	536	1
Kutty, et al. A. (2010)	USA	Patients for which there was no inpatient healthcare exposure within 8 weeks before the stool collection date	NR	*C. difficile* Tox A/B II ELISA	Veterans Affairs and Adults and elderly	NR	212	1
Norén, et al. (2004)	Sweden	Patients with community-acquired CDAD had no history of recent hospitalization	2,115	Antitoxin antibody neutralization, culture on cycloserine- cefoxitin-fructose agar	Mixed	274,000	59	1
Karlström, et al. (1998)	Sweden	Onset of symptoms outside the hospital without hospitalization within the preceding 4 weeks	NR	Culture (cycloserine-cefoxitin-fructose agar) and toxin A/B assay by immunologic	Mixed	8,800,000	89	1
van Dorp, et al. (2019)	Netherlands		12,714	NR	Mixed	2,810,830	124	1.33
Xia, et al. (2019)	Canada	CDI case with symptom onset in the community OR occurring less than or equal to 72 h or less than or equal to three calendar days after admission to a healthcare facility, provided that symptom onset was more than four weeks after the patient was discharged from any healthcare facility	NR	NR	NR	14,362,183	10,099	6
Lefevre-Tantet-Etchebarne, et al. (2016)	France	Acquired >12 weeks from hospitalization	2055	Glutamate dehydrogenase analysis by immunochromatographic test, and toxin analysis by immunochromatographic test and toxin-secreting strain culture	Adults and elderly	NR	28	3
Zilberberg, et al. (2016)	USA	If there was no evidence of an acute, nursing home, or skilled nursing facility stay within 12 weeks before the incident CDI episode or if there was no ICD–9-CM code for CDI	NR	NR	Elderly	1,165,165	1,197	2
Reigadas, et al. (2014)	Spain	Onset of symptoms while outside a healthcare facility, and without discharge from a healthcare facility within the previous 12 weeks or with onset of symptoms within 48 h following admission to a healthcare facility without residence in a healthcare facility within the previous 12 weeks	1,270	Toxigenic culture in Clostridium selective agar medium, immunochromatographic system and the MRC–5 cell line cytotoxicity test.	Mixed	NR	38	0.5
Marwick, et al. (2013)	Scotland	Onset of symptoms while outside a healthcare facility, and without discharge from a healthcare facility within the previous 12 weeks OR onset of symptoms within 48 h following admission to a healthcare facility, and without residence in a healthcare facility within the previous 12 weeks	NR	Toxin A & B test and/or the C. DIFF QUIK CHEK COMPLETETM test	Elderly	79,039	137	1
Hirschhorn, et al. (1994)	USA	Onset at least 42 days after the most recent hospitalization, CDAD with onset within 48 h of admission or CDAD in a patient admitted to a hospital with gastrointestinal symptoms and a positive assay for *C difficile* toxin within 5 days of admission	668	Cytotoxic assay as the confirmatory test for samples by latex agglutination.	Mixed	265,000	51	3
Asensio, et al. (2021)	Spain	Infections on patients with more than 4 weeks of discharge from a healthcare facility or with unknown origin	NR	Toxin A and B determination, PCR	Mixed	465,620	430	8
Zanichelli, et al. (2020)	Canada	Illness in a hospitalized patient for whom symptoms developed within 72 h of admission and who had not been hospitalized or received ambulatory care in the previous 4 weeks.	NR	Nucleic acid amplification tests and enzyme immunoassays for detecting toxigenic *C. difficile*	Mixed	8,209,599	4,481	7
Maisa, et al. (2019)	Ireland	No history of hospital admission >4 weeks from hospitalization; including cases with a positive CDI result 4–12 weeks of hospitalization OR < 48 h following hospital admission and no previous hospital stay within 4 weeks	2,807	Either glutamate dehydrogenase enzyme immunoassay or PCR testing, followed by toxin detection using ELISA	Mixed	1,866,042	1,303	5
Ho, et al. (2017)	China	Patients who had not been hospitalized in a healthcare facility within the previous 12 weeks	NR	Bacterial culture, toxin detection, NAAT	Adults and elderly	6,085,892	817	9
Abrahamian, et al. (2017)	USA	All cases that did not occur in participants with an overnight hospital or nursing home stay in the previous 3 months	422	*C. difficile*-positive culture result and positive toxin assay result either by the GeneXpert or ELISA	Mixed	NR	17	2.5
Kumar, et al. (2018)	Bailiwick of Jersey	Infection onset in the community with no prior admission to a healthcare facility for at least 84 days (12 weeks)	4,506	CD toxin enzyme immunoassay, glutamate dehydrogenase EIA	Mixed	99,000	29	5
Malmqvist, et al. (2019)	Sweden	Onset outside of healthcare facility and > 12 weeks since hospital discharge or onset within 48 h after admission to healthcare facility and > 12 weeks since hospital discharge	3,377	cell cytotoxicity assay using human embryonic lung fibroblasts, cytotoxicity neutralization assay for Toxin B and nucleic acid amplification test	Children	426,724	46	8
Russo, et al. (2019)	USA	Onset occurred in the community (outpatient setting) with no history of hospitalization, or long-term care, or skilled nursing facility stay during the previous 12 weeks	NR	Diagnosis code for CDI (ICD–9-CM 008.45 or ICD–10 A04.7 or the presence of toxin or toxin gene in a stool sample detected by enzyme immunoassay or polymerase chain reaction (PCR).	Adults	1,073,900	5,066	6
Weil, et al. (2007)	Germany	NR	703	enzyme immunoassay for *C. difficile* toxin A/B and culture	Mixed	NR	35	0.5
Furuya-Kanamori, et al. (2014)	Australia	Patient from the community that submitted a positive specimen for *C. difficile* toxin gene detection	24,496	tcdE and assay was agarose gel based or real time PCR with dual targets tcdE and tcdB	Mixed	4,500,000	1792	10
Yu, et al. (2022)	USA	Cases identified from the outpatient setting or an inpatient facility ≤3 days after admission, with no inpatient stay in the previous 12 weeks	NR	laboratory CDI test cell culture cytotoxicity assay, immunoassay, nucleic acid amplification	Elderly	6,100,000	61,470	8
Collins, et al. (2022)	Australia	NR	381	PCR result for tcdB, enzyme immunoassay for glutamate dehydrogenase (GDH), tcdB PCR	NR	2,200,000	112	3
Azimirad, et al. (2020)	Iran	Patients that developed CDI symptoms in the community or within 48 h or less after hospital admission. These patients must not have been discharged from a health-care facility in the previous 12 weeks	3,649	Cultured on cycloserine-cefoxitin-fructose agar, PCR on 16S rDNA gene	Mixed	NR	292	15
Younas, et al. (2019)	USA	Positive stool specimen was collected in an ambulatory setting or within three calendar days of hospital admission in a person with no documented overnight stay in a healthcare facility during the 12 weeks before the specimen was collected	NR	Nucleic-acid amplification test (NAAT), glutamate dehydrogenase test and enzyme immunoassays for toxin detection	Mixed	NR	5,192	1.5
Dantes, et al. (2015)	USA	Cases with no positive test ≤8 weeks prior and no overnight stay in a healthcare facility ≤12 weeks prior	NR	NAAT	Adults and elderly	10,400,000	4,682	1
Johnston, et al. (2021)	New Zealand	Cases with >12 weeks after discharge	1,153	Initial glutamate dehydrogenase screening assay, toxin enzyme immunoassay test	Mixed	NR	9	1
Guh, et al. (2020)	USA	Cases with no documented admission to a health care facility in the preceding 12 weeks	NR	NAAT	Mixed	12,000,000	47,512	7
Miranda, et al. (2020)	USA	*Cases evaluated* in the community or first 48 h of hospital admission and > 12 weeks after hospital discharge, with no prior positive *C difficile* testing in last 8 weeks, without other identified causes of diarrhea	7,650	Glutamate dehydrogenase antigen, PCR	Children	3,642,281	554	5
Bodé, et al. (2018)	Spain	Patient with CDI symptom onset in the community or 48 h or less after admission to a health care facility, provided that symptom onset was more than 12 weeks after the last discharge from an health care facility	7,004	Toxigenic culture on selective cycloserine cefoxitine fructose agar plates, Glutamate-dehydrogenase rapid enzyme immunoassay screening test was performed, rapid dual enzyme immunoassay screening test	Mixed	NR	151	2
Lasheras, et al. (2018)	Spain	Case of CDI with onset of symptoms: outside of healthcare facilities AND without discharge from a healthcare facility within the previous 12 weeks, or on the day of admission to a healthcare facility or on the following day and not resident in a healthcare facility within the previous 12 weeks	137	Toxigenic culture and enzyme immunoassay	Mixed	200,000	4	0.41
Na’amnih, et al. (2017)	Israel	Symptom onset in the community or within 48 h of admission to a hospital without any hospitalization in the previous 12 weeks and a positive stool sample within 48 h of admission	1,563	Enzyme immunoassay to detect toxin A/B, GDH and Toxin A/B immunochromatographic rapid test, CDT PCR	Adults and elderly	2,367,540	84	8
Garabasova, et al. (2017)	Slovakia	NR	NR	Epidemiological data, clinical picture (basic clinical symptoms and signs), microbiological tests (positive laboratory assay for *C. difficile* toxin A and/or B in stools)	Mixed	NR	51	0.25

NR: Not reported; CA-CDI: Community acquired *Clostridioides difficile* infection.

## Prevalence of CA-CDI

**Figure 2.**
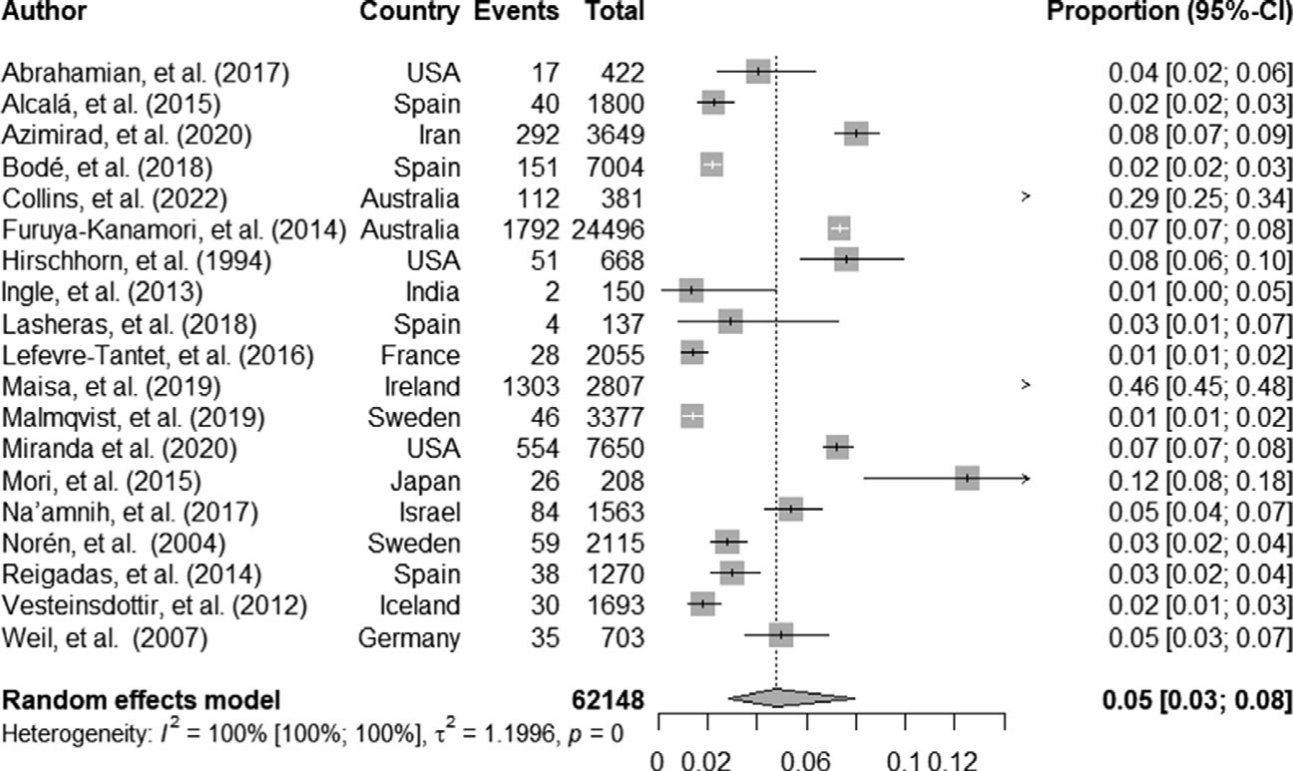
Pooled prevalence of CA-CDI.

The overall pooled prevalence of CA-CDI, obtained from a total of 62,148 patients, was 5% (95% CI 3–8; Figure 2). A subgroup analysis by age groups of the included samples from each study was performed, which showed no statistical subgroup difference (p = 0.58, Supplement III).

## Incidence of CA-CDI

**Figure 3.**
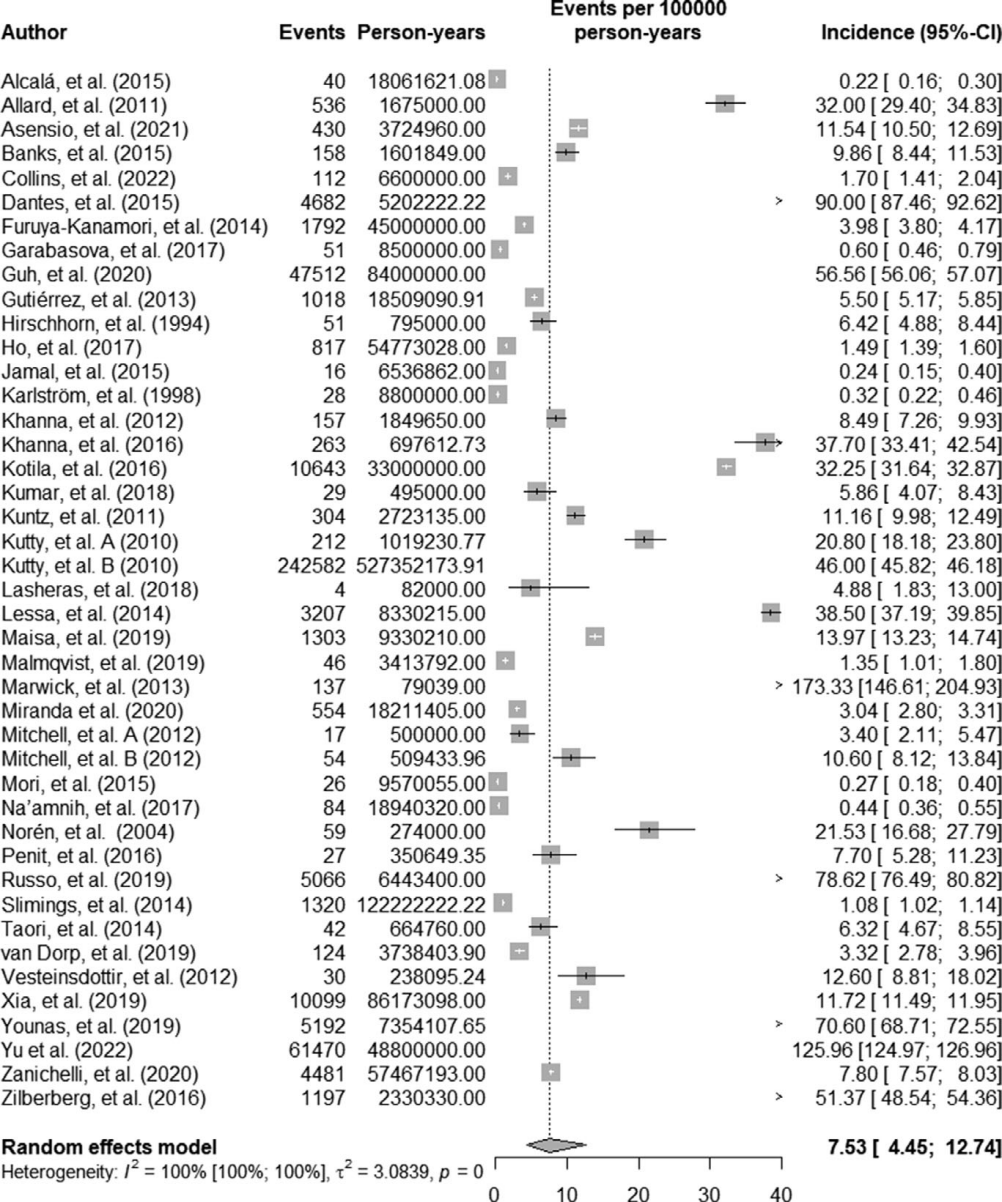
Pooled incidence of CA-CDI.

The overall pooled incidence of CA-CDI was 7.53 patients (95% CI 4.45–12.74) per 100,000 person-years (Figure 3). Furthermore, subgroup analysis revealed a statistically significant difference when divided by age group (p < 0.01, Supplement III).

## Heterogeneity and publication bias

This systematic review and meta-analysis detected high heterogeneity for both outcomes (I^2^ 100% 95% CI 100–100, p < 0.001). Preplanned sensitivity analysis was performed via influence analysis. For CA-CDI prevalence, influence analysis showed Maisa et al. [60] as a potential outlier, influencing the results. Repeating the analysis without Maisa et al. resulted in a pooled prevalence of 4% (95% CI 3–6), with a I^2^ of 98% (95% CI 98–98). On the other hand, for CA-CDI incidence, influence analysis did not show any potential outliers.

Publication bias was assessed visually and statistically via funnel plots and Egger’s test, respectively. Although the funnel plot for prevalence of CA-CDI visually showed asymmetry, Egger’s test did not indicate the presence of asymmetry (p = 0.1914). Publication bias of CA-CDI incidence showed a different result, with both the funnel plot and Egger’s test showing indication of publication bias (p = 0.0035). Both funnel plots can be seen in Supplementary material IV.

## Factors associated with CA-CDI

### Sex and gender

Several studies report an increase of CA-CDI cases in female patients. [28, 29] Furthermore, when compared to HA-CDI cases, CA-CDI patients were more likely to be female. [30, 46, 52] Other studies also found a statistically higher incidence of CA-CDI in females when compared to males [32], where Maisa et al. [60] reported that CA-CDI cases had lower odds to be male (adjusted odds ratio [aOR] 0.71; 95% CI 0.58–0.87; p < 0.001), but Ingle et al. reported that they did not find a statistically significant difference in gender. [58]

### Antibiotics

Antibiotic use has been identified before as a risk factor for *C. difficile* associated disease. [53, 64] Several studies reported that CA-CDI was more likely to have received antibiotics in the 2 months prior to developing the disease, with ORs ranging from 8.04–8.12 when compared to controls. [47, 62] Other authors have reported ORs of 6.09 (95% CI 4.59–8.08) when antibiotics were taken in the previous 6 months [31] and an almost 2-fold increased risk of CA-CDI when taking any antibiotic (1.94, 95% CI 1.35–2.77, p = 0.001). [48]

Some of the most commonly reported antibiotics associated with increased risk for CA-CDI were co-amoxicillin, fluoroquinolone, clindamycin and cephalosporins, fluoroquinolones, beta-lactam/beta-lactamase inhibitors, macrolides, and penicillins. [26, 27, 31, 48] Dantes et al. reported a predicted overall 16.8% (6.0%–26.3%; p = 0.003) decrease in CA-CDI incidence each 10% reduction in the use of all antibiotics. [26]

On the other hand, Ingle et al. [58] reported that although antibiotic use was more common in the CA-CDI group as compared to controls (66.7% vs. 38.4%, p = 0.07), the difference was not statistically significant, with other authors reporting similar results. [57] Nevertheless, when compared to HA-CDI, CA-CDI patients are less frequently taking antibiotics (p < 0.001) [37].

### Gastrointestinal therapy

Gastrointestinal therapy was also commonly evaluated as a risk factor for CA-CDI. Although Kuntz et al. [31] reported an aOR of 2.30 (95% CI 1.56–39) of developing CA-CDI when taking gastric acid suppressants 6 months before diagnosis, Jamal et al. reported a no-statistically significant prior exposure to gastrointestinal therapy when compared to control (p = 0.09). [63] Further more Mori et al. reported that prior exposure of antacids in the preceding 2 months was not a risk factor for CA-CDI (OR: 0.59, 95% CI: 0.19–1.85). [62]

Additionally, when compared to HA-CDI, CA-CDI patients less frequently received proton pump inhibitors (p < 0.001). [37]

### Age

Age was commonly reported as related to CA-CDI cases. Some studies have reported older age as a high predictor of CA-CDI cases, with different cut-off points, such as 40, 60, or 65 years [28, 29, 32, 51, 53] although not all studies have found the same difference. [58] Moreover, when compared to HA-CDI, CA-CDI cases were significantly younger. [30, 37, 60]

## Quality assessment

Out of 35 cross-sectional studies, 29 met the prespecified criteria for high quality study after being assessed using the AXIS tool, while 6 did not. [22, 39, 45, 46, 56, 66] The median score from the AXIS tool was 19. For case-control studies, assessed by the NOS, nine out of ten were of good/high quality, while one study was deemed as fair quality [32], with a median score of 7.5. All the four cohort studies included were of good/high quality, assessed using NOS, with a median score of 6. While these scores might be seen as abnormally high, it is important to consider that most studies included the total population and did not do any sampling.

## Discussion

The systematic review and meta-analysis conducted on 49 studies aimed to provide a comprehensive understanding of the prevalence and incidence of CA-CDI. The investigation involved a meticulous examination of a diverse range of literature, incorporating data from epidemiological studies conducted worldwide, allowing for a more nuanced and representative analysis, enhancing the reliability and generalizability of the results. This comprehensive approach not only contributes to the existing knowledge on CA-CDI but also offers valuable insights for healthcare professionals, researchers, and policymakers involved in the prevention and management of this infectious disease in community settings.

The cumulative prevalence of community-associated *C. difficile* infection was found to be 5%, significantly lower than the prevalence reported in the surveillance report by the eCDC. [67] According to their findings, 32.7% of cases recorded from 2016–2017 were attributed to community-associated CDI or CDI with an unknown origin of cases. Nevertheless, information regarding prior hospitalization was not consistently gathered for all cases. For those cases where such information was available, the duration of prior hospitalization varied from 4 to 12 weeks, potentially leading to misclassification, a limitation acknowledged by the authors. The same report states that it was twice as common for CA-CDI cases to report prior contact with a long-term care facility in the previous three months than for all CDI cases.

Our qualitative analysis revealed that prior antibiotic exposure emerged as a prominent risk factor for the onset of CA-CDI, aligning with the observations made by Deshpande et al. [68] Their study reported an OR of 6.91 (95% CI 4.17–11.44) for any antibiotic use. Notably, with the sole exception of tetracyclines, virtually all other classes of antibiotics exhibited an association with an elevated risk of CA-CDI.

Across all age groups and globally, the incidence of CDI was recorded at 7.5 cases per 100,000 person-years, nearly four times higher than the figure reported by Marwick and colleagues, which stood at 2.0. [48] However, in the context of adult patients, CA-CDI rates have been documented as reaching as high as 11.16 cases per 100,000 person-years. [31] It is worth noting that both referenced studies exclusively analyzed data from adults, unlike the compiled data, which includes a limited number of pediatric cases. Upon sub-analysis of the adult and elderly populations, the incidence escalates to 25 cases per 100,000 patient-years.

While CA-CDI can impact individuals of any gender, certain authors have documented a prevailing occurrence in females with a notable range spanning from 54% to 72.5%. [69, 70] However, this gender-based trend is not consistently observed across all studies.

There are several limitations of this systematic review and meta-analysis. Firstly, most of the prevalence results had to be calculated, rather than extracted, with data provided by the included studies. This was done because those papers reported CA-CDI proportions with the population being patients with CDI and not patients with diarrhea. Furthermore, although most of the cross-sectional studies met the criteria for high quality studies, most of them were population studies, inflating their AXIS score. Lastly, the funnel plot and Egger’s test showed some indication of publication bias on the incidence outcome; therefore, results should be interpreted cautiously.

It is relevant that most of the reports of CA-CDI include patients with already diagnosed *C. difficile*, not patients with diarrhea. Including this population directly compares the proportions of CA-CDI versus HA-CDI. If the proportion of CA-CDI increases the proportion of HA-CDI decreases and vice versa. Although this is useful to examine *C. difficile* behavior when evaluating community and hospital infections and onset, this may limit the understanding of the actual behavior of CDI in the community.

We highly recommend that, if the objective of the scientific community is to examine *C. difficile*, future studies should include patients with diarrhea as their population, not just patients with CDI. Further recommendations include reporting the exact number used as a denominator when calculating and reporting an incident.

## Conclusion

In conclusion, this meta-analysis underscores that among the included studies, the prevalence of CA-CDI stands at 5%, with an incidence rate of 7.3 cases per 100,000 person-years. Noteworthy risk factors identified include sex, prior antibiotic exposure, and age.

## Supporting information

Álvarez-Villalobos et al. supplementary material Álvarez-Villalobos et al. supplementary material

## Data Availability

All data relevant to the study are included in the article or uploaded as supplementary information.

## Supplementary material

For supplementary material accompanying this paper visit http://doi.org/10.1017/S0950268825000202. click here to view supplementary material

## References

[1] Stergachis A, et al. (1984)Antibiotic-associated colitis. The Western Journal of Medicine 140, 217–219. 6730468 PMC1021599

[2] McDonald LC, et al. (2007) Recommendations for surveillance of Clostridium difficile-associated disease. Infection Control and Hospital Epidemiology, 28, 140–145. 17265394 10.1086/511798

[3] Zanichelli V, et al. (2020) Increased community-associated Clostridioides difficile infections in Quebec, Canada, 2008–2015. Emerging Infectious Diseases Centers for Disease Control and Prevention 26, 1291. 10.3201/eid2606.190233 PMC7258478 32441632

[4] Guh AY, et al. (2020) Trends in U.S. burden of Clostridioides difficile infection and outcomes. The New England Journal of Medicine 382, 1320–1330. 32242357 10.1056/NEJMoa1910215 PMC7861882

[5] Gravel D, et al. (2009) Health care-associated Clostridium difficile infection in adults admitted to acute care hospitals in Canada: A Canadian Nosocomial Infection Surveillance Program Study. Clinical Infectious Diseases: An Official Publication of the Infectious Diseases Society of America 48, 568–576. 19191641 10.1086/596703

[6] Joshi NM, Macken L, Rampton DS (2012) Inpatient diarrhoea and Clostridium difficile. Clinical Medicine Royal College of Physicians 12, 583. 23342416 10.7861/clinmedicine.12-6-583 PMC5922602

[7] Khanna S, et al. (2012) Outcomes in community-acquired Clostridium difficile infection. Alimentary Pharmacology and Therapeutics John Wiley & Sons, Ltd 35, 613–618. 22229532 10.1111/j.1365-2036.2011.04984.x PMC3293482

[8] Kotila SM, et al. (2016) Community- and healthcare-associated Clostridium difficile infections, Finland, 2008−2013. Emerging Infectious Diseases Centers for Disease Control and Prevention 22, 1747–1753. 27648884 10.3201/eid2210.151492 PMC5038409

[9] Levy AR, et al. (2015) Incidence and Costs of *Clostridium difficile* Infections in Canada; Published online: 2015. 10.1093/ofid/ofv076. PMC4503917 26191534

[10] Zhang D, Prabhu VS, Marcella SW (2018) Attributable healthcare resource utilization and costs for patients with primary and recurrent Clostridium difficile infection in the United States. Clinical infectious diseases: an official publication of the Infectious Diseases Society of America 66, 1326–1332. 29360950 10.1093/cid/cix1021 PMC5905590

[11] Dubberke ER, Olsen MA (2012) Burden of Clostridium difficile on the healthcare system. Clinical infectious diseases: an official publication of the Infectious Diseases Society of America 55 (Suppl 2)Published online: 1 August 2012. 10.1093/CID/CIS335. PMC3388018 22752870

[12] Bloomfield LE, Riley TV (2016) Epidemiology and risk factors for community-associated Clostridium difficile infection: A narrative review. Infectious Diseases and Therapy, Springer 5, 231. 27370914 10.1007/s40121-016-0117-y PMC5019973

[13] Du T, et al. (2022) Characterization of healthcare-associated and community-associated Clostridioides difficile infections among adults, Canada, 2015–2019. Emerging Infectious Diseases Centers for Disease Control and Prevention 28, 1128. 35470794 10.3201/eid2806.212262 PMC9155897

[14] Khanna S, Pardi DS (2010)The growing incidence and severity of Clostridium difficile infection in inpatient and outpatient settings. Expert Review of Gastroenterology and Hepatology 4, 409–416. 20678014 10.1586/egh.10.48

[15] Ofori E, et al. (2018) Community-acquired Clostridium difficile: Epidemiology, ribotype, risk factors, hospital and intensive care unit outcomes, and current and emerging therapies. Journal of Hospital Infection Elsevier Ltd 99, 436–442. 29410012 10.1016/j.jhin.2018.01.015

[16] Tenny S, Boktor SW (2023) *StatPearls: Incidence. StatPearls*. (https://www.ncbi.nlm.nih.gov/books/NBK430746/). 28613497

[17] Downes MJ, et al. (2016) Development of a critical appraisal tool to assess the quality of cross-sectional studies (AXIS). BMJ Open BMJ Publishing Group 6 Published online: 1 December 2016. 10.1136/BMJOPEN-2016-011458. PMC5168618 27932337

[18] Wells G, et al. (2011) *The Newcastle-Ottawa Scale (NOS) for Assessing the Quality of Nonrandomised Studies in meta-Analyses*. Ottawa: University of Ottawa.

[19] Stijnen T, Hamza TH, Özdemir P. (2010) Random effects meta-analysis of event outcome in the framework of the generalized linear mixed model with applications in sparse data. Statistics in Medicine 29, 3046–3067. 20827667 10.1002/sim.4040

[20] Cumpston M, et al. (2019) Updated guidance for trusted systematic reviews: A new edition of the Cochrane handbook for systematic reviews of interventions. The Cochrane database of systematic reviews, NLM (Medline) 10, ED000142. 31643080 10.1002/14651858.ED000142 PMC10284251

[21] Higgins JPT, Thompson SG (2002) Quantifying heterogeneity in a meta-analysis. Statistics in Medicine 21, 1539–1558. 12111919 10.1002/sim.1186

[22] Khanna S, et al. (2016) Tu1070 changing epidemiology of Clostridium difficile from predominantly hospital-acquired to community-acquired infection. Gastroenterology AGA Institute 150, S832–S833.

[23] Russo EM, et al. (2019) Incidence of Clostridioides difficile infections among young and middle-aged adults: Veterans health administration. Infection Control and Hospital Epidemiology 40, 997–1005. 31322101 10.1017/ice.2019.160

[24] Yu H, et al. (2022) Incidence, attributable mortality, and healthcare and out-of-pocket costs of Clostridioides difficile infection in US Medicare advantage enrollees. Clinical Infectious Diseases Oxford University Press 76, E1476–E1483. 10.1093/cid/ciac467 PMC9907506 35686435

[25] Younas M, et al. (2019) Burden of community-associated Clostridioides difficile infection in southeastern United States: A population-based study. Infection Springer Berlin Heidelberg 48, 129–132. 31677084 10.1007/s15010-019-01368-5

[26] Dantes R, et al. (2015) Association between outpatient antibiotic prescribing practices and community-associated Clostridium difficile infection. Open Forum Infectious Diseases 2, 2633851. 10.1093/ofid/ofv113 PMC4551478 26509182

[27] Miranda-Katz M, et al. (2020) Epidemiology and risk factors for community associated Clostridioides difficile in children. Journal of Pediatrics Elsevier Inc 221, 99–106. 32171559 10.1016/j.jpeds.2020.02.005

[28] Lessa FC, et al. (2014) Determinants of Clostridium difficile infection incidence across diverse United States geographic locations. Open Forum Infectious Diseases 1, 2633851. 10.1093/ofid/ofu048 PMC4281776 25734120

[29] Gutiérrez RL, Riddle MS, Porter CK (2013) Epidemiology of clostridium difficile infection among active duty United States military personnel (1998-2010). BMC Infectious Diseases 13 Published online: 2013. 10.1186/1471-2334-13-609. PMC3880161 24373384

[30] Khanna S, et al. (2012) The epidemiology of community-acquired clostridium difficile infection: A population-based study. American Journal of Gastroenterology Nature Publishing Group 107, 89–95. 22108454 10.1038/ajg.2011.398 PMC3273904

[31] Kuntz JL, et al. (2011) Incidence of and risk factors for community-associated Clostridium difficile infection: A nested case-control study. BMC Infectious Diseases BioMed Central Ltd 11, 194. 21762504 10.1186/1471-2334-11-194 PMC3154181

[32] Kutty PK, et al. (2010) Risk factors for and estimated incidence of community-associated Clostridium difficile infection, North Carolina, USA. Emerging Infectious Diseases 16, 197–204. 20113547 10.3201/eid1602.090953 PMC2958012

[33] Zilberberg MD, et al. (2016) Development and validation of a risk score for Clostridium difficile infection in Medicare beneficiaries: A population-based cohort study. Journal of the American Geriatrics Society 64, 1690–1695. 27295521 10.1111/jgs.14236

[34] Hirschhorn LR, et al. (1994) Epidemiology of community-acquired Clostridium difficile-associated diarrhea. Journal of Infectious Diseases 169, 127–133. 8277174 10.1093/infdis/169.1.127

[35] Abrahamian FM, et al. (2017) Clostridium difficile infection among US emergency department patients with diarrhea and no vomiting. Annals of Emergency Medicine 70, 19–27.e4. 28242058 10.1016/j.annemergmed.2016.12.013

[36] Alcalá L, et al. (2015) Impact of clinical awareness and diagnostic tests on the underdiagnosis of Clostridium difficile infection. European Journal of Clinical Microbiology and Infectious Diseases 34, 1515–1525. 25904126 10.1007/s10096-015-2380-3

[37] Reigadas E, et al. (2014) Missed diagnosis of Clostridium difficile infection; a prospective evaluation of unselected stool samples. Journal of Infection 70, 264–272. 25452039 10.1016/j.jinf.2014.10.013

[38] Asensio Á, et al. (2022) Epidemiology of Clostridioides difficile infection in hospitalized patients in Spain: An eight-year review (2012–2019). Enfermedades Infecciosas y Microbiología Clínica 40, 125–130. 35249672 10.1016/j.eimce.2021.04.008

[39] Suárez-Bode L, et al. (2018) Increasing prevalence of the epidemic ribotype 106 in healthcare facility–associated and community-associated Clostridioides difficile infection. Anaerobe 55, 124–129. 30550807 10.1016/j.anaerobe.2018.12.002

[40] Andrés-Lasheras S, et al. (2018) Incidence and characterization of Clostridium difficile in a secondary care hospital in Spain. Revista Espanola De Enfermedades Digestivas 111, 338–344. 10.17235/reed.2018.5288/2017 30569726

[41] Slimings C, et al. (2014) Increasing incidence of Clostridium difficile infection, Australia, 2011-2012. Medical Journal of Australia 200, 272–276. 24641152 10.5694/mja13.11153

[42] Mitchell BG, Rn FW, Mcgregor A. (2012) *An Increase in Community Onset Clostridium Difficile Infection: A Population-Based Study*, Tasmania, Australia 127–132.

[43] Furuya-Kanamori L, et al. (2014) A population-based spatio-temporal analysis of Clostridium difficile infection in Queensland, Australia over a 10-year period. Journal of Infection 69, 447–455. 24984276 10.1016/j.jinf.2014.06.014

[44] Xia Y, et al. (2019) Epidemiology of Clostridioides difficile infection in Canada: A six-year review to support vaccine decision-making. Canada Communicable Disease Report 45, 191–211. 31355824 10.14745/ccdr.v45i78a04 PMC6615439

[45] Allard R, et al. (2011) Community-acquired Clostridium difficile – associated diarrhea, Montréal, 2005–2006: Frequency estimates and their validity. Infection Control and Hospital Epidemiology 32, 1032–1034. 21931255 10.1086/661911

[46] Banks A, et al. (2015) Sentinel community Clostridium difficile infection (CDI) surveillance in Scotland, April 2013 to March 2014. Anaerobe Elsevier Ltd 37, 49–53. 26708405 10.1016/j.anaerobe.2015.12.008

[47] Taori SK, et al. (2014) A prospective study of community-associated Clostridium difficile infections: The role of antibiotics and co-infections. Journal of Infection Elsevier Ltd 69, 134–144. 24780765 10.1016/j.jinf.2014.04.002

[48] Marwick CA, et al. (2013) Community-associated Clostridium difficile infection among older people in Tayside, Scotland, is associated with antibiotic exposure and care home residence: Cohort study with nested case-control. Journal of Antimicrobial Chemotherapy 68, 2927–2933. 23825381 10.1093/jac/dkt257

[49] Malmqvist L, et al. (2019) Clostridium difficile infection in children: Epidemiology and trend in a swedish tertiary care hospitalt. Pediatric Infectious Disease Journal 38, 1208–1213. 31738336 10.1097/INF.0000000000002480

[50] Norén T, et al. (2004) Molecular epidemiology of hospital-associated and community-acquired Clostridium difficile infection in a Swedish county. Journal of Clinical Microbiology 42, 3635–3643. 15297509 10.1128/JCM.42.8.3635-3643.2004 PMC497655

[51] Karlström O, et al. (1998) A prospective nationwide study of Clostridium difficile-associated diarrhea in Sweden. Clinical Infectious Diseases 26, 141–145. 9455523 10.1086/516277

[52] Penit A, et al. (2016) Community-acquired Clostridium difficile infections. Medecine et Maladies Infectieuses Elsevier Masson SAS 46, 131–139. 27039068 10.1016/j.medmal.2016.01.007

[53] Lefevre-Tantet-Etchebarne D, et al. (2016) Infections communautaires à Clostridium difficile dans les services d’urgence. Medecine et Maladies Infectieuses Elsevier Masson SAS 46, 372–379. 27377443 10.1016/j.medmal.2016.06.002

[54] Kumar S, et al. (2018) The epidemiology of community clostridium difficile infection: A five-year population-based study on the bailiwick of Jersey, Channel Islands. Infection Control and Hospital Epidemiology 39, 603–607. 29485017 10.1017/ice.2018.38

[55] Ho J, et al. (2017) Disease burden of Clostridium difficile infections in adults, Hong Kong, China, 2006–2014. Emerging Infectious Diseases 23, 1671–1679. 28930010 10.3201/eid2310.170797 PMC5621553

[56] Weil HP, et al. (2007) O329 High incidence of Clostridium difficile associated diarrhoea with a community onset in a hyperendemic region in Germany. International Journal of Antimicrobial Agents 29, S69.

[57] Vesteinsdottir I, et al. (2012) Risk factors for clostridium difficile toxin-positive diarrhea: A population-based prospective case-control study. European Journal of Clinical Microbiology and Infectious Diseases 31, 2601–2610. 22441775 10.1007/s10096-012-1603-0

[58] Ingle M, et al. (2013) Clostridium difficile as a cause of acute diarrhea: A prospective study in a tertiary care center. Indian Journal of Gastroenterology 32, 179–183. 23526401 10.1007/s12664-013-0303-8

[59] Azimirad M, et al. (2020) Clostridioides difficile ribotypes 001 and 126 were predominant in Tehran healthcare settings from 2004 to 2018: A 14-year-long cross-sectional study. Emerging Microbes and Infections 9, 1432–1443. 32520657 10.1080/22221751.2020.1780949 PMC7473134

[60] Maisa A, et al. (2019) Comparing the epidemiology of community- and hospital-associated clostridium difficile infections in northern Ireland, 2012–2016: A population data linkage and case–case study. Epidemiology and Infection 147, 2012–2016. 10.1017/S0950268819000414 PMC6518519 30869054

[61] Na’Amnih W, et al. (2017) Incidence and risk factors for community and hospital acquisition of Clostridium difficile infection in the Tel Aviv Sourasky Medical Center. Infection Control and Hospital Epidemiology 38, 912–920. 28558856 10.1017/ice.2017.82

[62] Mori N, Aoki Y (2015) Clinical characteristics and risk factors for community-acquired Clostridium difficile infection: A retrospective, case-control study in a tertiary care hospital in Japan. Journal of Infection and Chemotherapy Elsevier Ltd 21, 864–867. 10.1016/j.jiac.2015.09.004 26482373

[63] Jamal W, Pauline E, Rotimi V (2015) A prospective study of community-associated Clostridium difficile infection in Kuwait: Epidemiology and ribotypes. Anaerobe Elsevier 35, 28–32. 10.1016/j.anaerobe.2015.06.006 26144314

[64] van Dorp SM, et al. (2019) Spatial clustering and livestock exposure as risk factor for community-acquired Clostridium difficile infection. Clinical Microbiology and Infection Elsevier Ltd 25, 607–612. 10.1016/j.cmi.2018.07.018 30076972

[65] Johnston M, et al. (2022) Clostridioides difficile infection in a rural New Zealand secondary care Centre: An incidence case–control study. Internal Medicine Journal 52, 1009–1015. 33528096 10.1111/imj.15220

[66] Garabasova MK, et al. (2017) Burden of clostridium difficile infection among hospitalized patients in Slovakia. Antimicrobial Resistance and Infection Control 6, 32.28360994

[67] The European Surveillance System (2017) Clostridioides (Clostridium) difficile infections - Annual Epidemiological Report for 2016–2017.

[68] Deshpande A, et al. (2013) Community-associated Clostridium difficile infection and antibiotics: A meta-analysis. Journal of Antimicrobial Chemotherapy Oxford Academic 68, 1951–1961. 23620467 10.1093/jac/dkt129

[69] Bauer MP, et al. (2009) Clinical and microbiological characteristics of community-onset Clostridium difficile infection in the Netherlands. Clinical Microbiology and Infection 15, 1087–1092. 19624512 10.1111/j.1469-0691.2009.02853.x

[70] Thornton CS, et al. (2018) Epidemiological and genomic characterization of community-acquired Clostridium difficile infections. BMC Infectious Diseases 18, 443. 30170546 10.1186/s12879-018-3337-9 PMC6119286

[71] Collins DA, et al. (2022) Linkage study of surveillance and hospital admission data to investigate Clostridium difficile infection in hospital patients in Perth, Western Australia. Anaerobe Elsevier Ltd 74, 102528. 10.1016/j.anaerobe.2022.102528 35104667

